# Seroprevalence of Toxoplasma gondii Infection in Pet Dogs in Anhui Province, China

**DOI:** 10.18502/ijpa.v15i3.4211

**Published:** 2020

**Authors:** Zhijin SHENG, Yu JIN, Yong YAO, Saeed El-ASHRAM, Jijia SHEN, Xue-long WANG, Yongsheng JI

**Affiliations:** 1. Department of Physical Education, College of Humanistic Medicine, Anhui Medical University, Hefei, Anhui, China; 2. Anhui Provincial Laboratory of Pathogen Biology, Anhui Medical University, Hefei, Anhui, China; 3. Anhui Key Laboratory of Zoonoses, Anhui Medical University, Hefei, Anhui, China; 4. Department of Microbiology and Parasitology, Anhui Medical University, Hefei, Anhui, China; 5. College of Life Science and Engineering, Foshan University, Foshan, Guangdong Province, China; 6. Department of Immunology and Parasitology, Faculty of Science, Kafrelsheikh University, Egypt

**Keywords:** *Toxoplasma gondii*, Dogs, Modified agglutination test, Seroprevalence, China

## Abstract

**Background:**

*Toxoplasma gondii* is an obligate intracellular parasite, which can infect all nucleated cells in a variety of vertebrate animals, including human, causing toxoplasmosis. Although a number of studies have reported on the seroprevalence of *T. gondii* infection in dogs in China, however, information about *T. gondii* infection in pet dogs in Anhui, China is not available.

**Methods:**

The modified agglutination test (MAT) was used to detect antibodies in sera samples from 468 pet dogs at Anhui Province in China from November 2013 to April 2017.

**Results:**

18.6% animals were *T. gondii* seropositive, indicating a slightly higher prevalence of *T. gondii* infection in pet dogs in Anhui, China in comparison with other provinces in China.

**Conclusion:**

Our present study provided epidemiological data on *T. gondii* seroprevalence in pet dogs in Anhui, China for the effective prevention and control of the parasite prevalence in this area.

## Introduction

*Toxoplasma gondii* is an obligate intra-cellular parasite, which infects all nucleated cells in humans and a broad spectrum of vertebrate hosts, leading to zoonosis termed toxoplasmosis (1). About one third of the human population was chronically infected with this parasite, and most of them were asymptomatic (2).

Currently, little national data has been available on the chronic infection rate of *T. gondii* in China, and a large-scale serological survey of *T. gondii* infection among persons participated in health screening demonstrated that the total seroprevalence of *T. gondii* infection was 6.67% in Yunnan Province, Southwestern China (3).

Human can be infected by eating under-cooked meat containing tissue cysts or oocyst contaminated food and water. Moreover, *Toxoplasma* can be transmitted congenitally, and has been associated with severe disease, including toxoplasmic encephalitis, blindness, abortion, fetal abnormalities, and even prenatal death could be caused in *Toxoplasma-*infected pregnant women were infected with *T. gondii* (4). *Toxoplasma* is an opportunistic pathogen, which can be lethal for immuno-compromised patients (e.g. HIV/AIDS patients) (5).

With the current economic and social development of China, pet ownership has increased in China, and dogs are the most preferred pets. However, previous studies have shown that dogs may be involved in the mechanical transmission of a number of zoonotic diseases, including *T. gondii* to humans (6). Thus, it is important to survey the seroprevalence of *T. gondii* infection in dogs and assess to what extent dogs constitute a reservoir and source of infection for humans. A number of studies have been reported about the *T. gondii* infection in dogs in China over the last 10 years (7–11), but only limited surveys of *T. gondii* infection in dogs in Anhui Province are available (12).

The aim of present study was to investigate the seroprevalence of *T. gondii* infection in pet dogs in Anhui province of China.

## Materials and Methods

### Ethics statement

The present study was approved by the Animal Ethics Committee of Anhui Medical University (Permit code AhMU20130086). Dogs from which sera samples were collected were handled in accordance with the Animal Ethics Procedures and Guidelines of the People’s Republic of China.

### The site

**Fig. 1:**
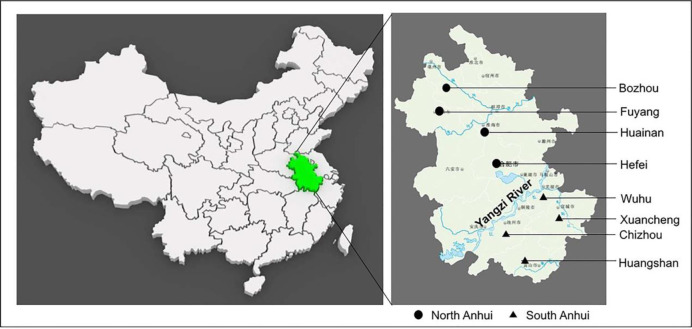
Geographic location of the present survey

Anhui province is located at Central China, covering an area of 140,100 km ^2^ and a population of approximately 70.7 million people (Fig. 1).

Anhui is situated between east longitudes of 114°54’ to 119°37’and north latitudes of 29°41’ to 34°38’. It has a typical subtropical monsoon climate, with an annual rainfall of 773–1670 mm, and an average annual temperature of 14–17°C. The average annual rainfall of Anhui is 773–1, 670 mm, and the average annual temperature is 14–17 °C, both of which are suitable conditions for the development of *T. gondii* oocysts in environments.

### Collection and preparation of serum samples

Our survey was conducted between November 2013 and April 2017. A total of 468 blood samples were collected from pet dogs. These pet dogs were admitted into pet hospitals located in eight cities of Anhui Province, namely Hefei, Huainan, Fuyang, Bozhou, Wuhu, Xuancheng, Huangshan and Chizhou. Information regarding the ages and genders of the pet dogs was provided by dog owners. For each dog, at least 3 ml of blood was drawn by jugular venipuncture into a syringe. Blood samples were transferred to tubes and kept at 37 °C for 2 h (h), centrifuged at 2,000 g for 5 min, and the sera samples were stored at −20 °C until further processing.

### Serological examinations

The modified agglutination test (MAT), a sensitive and specific technique was used for detecting *T. gondii* antibodies (13). Briefly, a suspension of *Toxoplasma* tachyzoites was prepared and fixed with formalin. Sera samples were diluted in phosphate buffered saline (PBS, pH 7.2). Other materials used in this study included positive and negative control sera (kindly provided from Jilong Shen group, Anhui Medical University), antigen diluting buffer containing bovine serum albumin (BSA), 2-mercaptoethanol, and Evans blue dye solution. MAT titers of 1:20 or higher were considered as positive (14), and those sera with dubious results were re-tested. Positive and negative controls were incorporated in each test and investigated at the same dilutions of serum samples.

### Statistical analysis

Differences in the seroprevalence of *T. gondii* infection between male and female dogs, and among different age groups were analyzed using a Chi square test using the SPSS (Release 20.0 standard version, SPSS Inc., Chicago, Illinois). Differences were considered statistically significant at *P* < 0.05.

## Results

Out of 468 pet dogs in Anhui Province, 87 (18.6%, 95% CI 15.1~22.1) were seropositive for *T. gondii* (Table 1). Antibody titers of 1:20 were found in 42 dogs, 1:40 in 31, 1:80 in 14, while 1:160 or higher was not detected.

**Table 1:** Prevalence of *Toxoplasma gondii* in pet dogs in Anhui Province, China

***Cities***	***No. of sera with MAT titers of***						***No. examined***	***No. positive***	***Prevalence (%, 95% CI)***

	1:5	1:10	1:20	1:40	1:80	1:160			
Bozhou	0	0	5	4	0	0	58	9	8.6 (5.9–25.1)
Fuyang	0	0	4	4	3	0	65	11	16.9 (7.6–26.3)
Huainan	0	0	4	3	0	0	47	7	14.9 (4.3–25.5)
Hefei	0	0	5	2	2	0	56	9	16.1 (6.1–26.0)
Chizhou	0	0	6	4	4	0	58	14	24.1 (12.8–35.5)
Wuhu	0	0	7	4	3	0	66	14	21.2 (11.1–31.3)
Huangshan	0	0	6	5	1	0	61	12	19.7 (9.4–29.9)
Xuancheng	0	0	5	5	1	0	57	11	19.3 (8.7–29.9)
Total	0	0	42	31	14	0	468	87	18.6 (15.1–22.1)

The variables associated with *T. gondii* sero-prevalence in pet dogs were also analyzed in present study. We found that the seroprevalence of *T. gondii* infection in males was slightly higher than that in females (Table 2, 21.7% vs 15.9%, *P*=0.142). Meanwhile, the seroprevalence of *T. gondii* in pet dogs in North Anhui was lower than that in South Anhui (Table 2, 15.9% vs 21.1%, P=0.190).

**Table 2:** Analysis of the variables associated with *Toxoplasma gondii* seroprevalence in Anhui, China

***Variable***		***No. examined***	***No. positive***	***Prevalence (%, 95% CI)***	***P value***
Locations	North Anhui	226	36	15.9 (11.1–20.7)	0.190
	South Anhui	242	51	21.1 (15.9–26.2)	
Gender	Male	214	48	21.7 (16.8–28.1)	0.142
	Female	253	39	15.9 (10.9–19.9)	
Age (yr)	<1	102	15	14.7 (7.7–21.7)	
	1–2	122	24	19.7 (12.5–26.8)	
	2–3	124	22	17.7 (10.9–24.6)	
	>3	120	26	21.7 (14.2–29.1)	

## Discussion

Our present study provided epidemiological data on *T. gondii* seroprevalence in pet dogs in Anhui, demonstrating that 18.6% animals involved in our study were *T. gondii* seropositive. The overall seroprevalence of *T. gondii* in the present study was lower than that observed in pet dogs of Beijing (23.1 %) (15), Kunming (21.6 %) (10), Central China (24 %) (14) and slaughter dogs in Jilin, Anhui and Henan (8.24 %) (12) and police dogs in Shenyang (30.9 %) (16), but were higher than those of Shanghai (3.2 %) (17) and pet dogs in Shenyang (10.0 %) (11), Guizhou (0.93 %) (18), Sichuan (3.5%) (19), Lanzhou (10.81 %) (9). A seroprevalence survey showed that *T. gondii* seropositive rate in stray dogs (18.5%) was higher than that of pet dogs (10.0%) in Shandong, Henan, Heilongjiang and Xinjiang (20). These differences may be due to different serological testing methods, survey periods, pet welfare conditions, climate and geographical factors in these areas.

Although there was no significant difference among age groups, the seroprevalence of toxoplasmosis in pet dogs increased progressively with age (Table 2, except 2–3 years group), indicating that the exposure of pet dog to *T. gondii* oocysts in the environments or tissue cysts in meat may contribute to the spread of toxoplasmosis. A large-scale investigation is needed to understand why seroprevalence of *T. gondii* infection in pet dogs aged 2–3 years was lower.

Consistent with results from previous study (16), the seroprevalence of *T. gondii* infection in males was slightly higher than that in females. This may be ascribed to male dogs having more opportunities to come into contact with environments, which may be contaminated with oocysts of *T. gondii*. Anhui province is geographically divided by Yangzi River into two parts, North Anhui and South Anhui (Fig. 1). These two parts are different in climate. The climate is dry in North Anhui area, while South Anhui is moist, which is more suitable for *T. gondii* oocyst development. This may explain the difference between seroprevalence of *T. gondii* in pet dogs in North Anhui and that in South Anhui.

## Conclusion

The overall seroprevalence of *T. gondii* in pet dogs in Anhui Province, China was 18.6%. Our study provides baseline data for the effective prevention and control of the parasite prevalence in this area. Further studies will be conducted to isolate *T. gondii* strain from infected dogs and determine the genotypes of *T. gondii* infected dogs in Anhui area.
